# Perinatal Outcomes in HIV Positive Pregnant Women with Concomitant Sexually Transmitted Infections

**DOI:** 10.1155/2015/508482

**Published:** 2015-03-31

**Authors:** Erin Burnett, Tammy L. Loucks, Michael Lindsay

**Affiliations:** Department of Gynecology and Obstetrics, Emory University School of Medicine, 69 Jesse Hill Jr. Drive SE, Glenn Building, 4th Floor, Atlanta, GA 30303, USA

## Abstract

*Objective*. To evaluate whether HIV infected pregnant women with concomitant sexually transmitted infection (STIs) are at increased risk of adverse perinatal and neonatal outcomes.* Methods*. We conducted a cohort study of HIV positive women who delivered at an inner-city hospital in Atlanta, Georgia, from 2003 to 2013. Demographics, presence of concomitant STIs, prenatal care information, and maternal and neonatal outcomes were collected. The outcomes examined were the association of the presence of concomitant STIs on the risk of preterm birth (PTB), postpartum hemorrhage, chorioamnionitis, preeclampsia, intrauterine growth restriction, small for gestational age, low Apgar scores, and neonatal intensive care admission. Multiple logistic regression was performed to adjust for potential confounders.* Results*. HIV positive pregnant women with concomitant STIs had an increased risk of spontaneous PTB (odds ratio (OR) 2.11, 95% confidence interval [CI] 1.12–3.97). After adjusting for a history of preterm birth, maternal age, and low CD4+ count at prenatal care entry the association between concomitant STIs and spontaneous PTB persisted (adjusted OR 1.96, 95% CI 1.01–3.78).* Conclusions*. HIV infected pregnant women with concomitant STIs relative to HIV positive pregnant women without a concomitant STI are at increased risk of spontaneous PTB.

## 1. Introduction

Human immunodeficiency virus (HIV) infections continue to increase in the United States. As of 2012 an estimated 1.3 million people living in the United States are HIV positive (0.6% of USA population) [1]. According to data from the US Global Health policy released in December 2012, there are 50,000 new HIV infections yearly in the United States. HIV infections among women, especially those of reproductive age, have also dramatically increased in the past 2 decades [1]. Further, observational studies conducted in the United States have documented a link between HIV infection and adverse pregnancy outcomes including low birth weight (LBW), preterm birth (PTB), and small for gestational age (SGA) infants [2–6].

The prevalence of sexually transmitted infections (STIs) in the United States is increasing and the majority of these infections occur in women of reproductive age. A 2007 study estimated that 15 million new STIs occur annually and that 13.3% of these, about 2 million, occur in pregnant women [7]. In 2011 the Centers for Disease Control and Prevention (CDC) reported that the overwhelming majority of all infections in females occurred in women of reproductive age [8]. Multiple observational studies performed in the United States have linked selected STIs, including gonorrhea, chlamydia, trichomoniasis, human papillomavirus (HPV), syphilis, and herpes simplex virus, to poor perinatal and neonatal outcomes including, intrauterine growth restrictions (IUGR), preterm labor (PTL), preterm birth (PTB), premature rupture of membranes (PROM), chorioamnionitis, LBW, and postpartum endometritis [9–13].

Current evidence supports a link between HIV infection and adverse perinatal outcomes as well as a link between STIs and adverse perinatal outcomes. However, there is a paucity of data on perinatal outcomes in HIV positive women with concomitant STIs.

To address this knowledge gap we identified a cohort of HIV infected pregnant women who received prenatal care and delivered at an inner-city hospital. We examined the hypothesis that concomitant STIs would be associated with an increased risk for adverse perinatal and neonatal outcomes in this HIV positive cohort.

## 2. Methods

A cohort of all HIV infected women who delivered at Grady Memorial Hospital (GMH) over a span of 10.5 years was identified. GMH is an inner city hospital which provides care for indigent patients throughout the metropolitan Atlanta area, the majority of which are minorities. HIV infected women were followed during pregnancy in a HIV specific obstetrical clinic run by the maternal fetal medicine fellows under the direction of the same fellowship director during the entire study period. Treatment protocol for HIV was in accordance with the most up to date National Institute of Health (NIH) recommendations at the time. Every effort is made for STIs treatment immediately upon diagnosis. This study received approval from the Institutional Review Board, at Emory University and the Grady Memorial Hospital Research Oversight Committee (IRB study # 00064558). The medical and delivery records of all HIV positive women with singleton pregnancies delivered at GMH from January 1, 2003, through May 31, 2013, were reviewed. Pregnancies ending in a spontaneous or therapeutic abortion at <20 weeks were excluded, along with women who did not receive prenatal care at GMH.

Demographic and clinical data extracted from the records included age, race, weight (at first prenatal visit), gravidity, parity, and history of preterm delivery (<37 weeks). HIV related information extracted included year of HIV diagnosis, whether or not the diagnosis was with the current pregnancy, duration, and compliance with antiretroviral therapy, viral genotype, viral load, and CD4 counts at the beginning of prenatal care and near delivery. In addition, types and number of occurrences of STIs diagnosed during the pregnancy were recorded. Testing for syphilis, HPV (Pap), chlamydia, and gonorrhea was done at entry to care and then anytime a patient had presence of symptoms a wet prep and chlamydia and gonorrhea was performed. Third trimester screening for syphilis, chlamydia, and gonorrhea was done per routine prenatal care. STIs of interest included chlamydia, gonorrhea, herpes simplex (HSV), syphilis, trichomoniasis, and HPV. Chlamydia and gonorrhea were diagnosed by DNA probes; herpes simplex virus was diagnosed by culture, physician exam, or patient reported symptoms in women with known history of HSV; syphilis was diagnosed via positive serology titers, trichomoniasis was diagnosed via wet prep, and HPV infection was based on the presence of abnormal cervical cytology. Perinatal outcomes examined included the number of antepartum admissions, rate of intrauterine growth restriction, preeclampsia, chorioamnionitis, anemia, preterm labor, abruption, intrauterine fetal demise (IUFD), spontaneous and indicated preterm delivery (<37 weeks), and preterm premature rupture of membranes (PROM). Delivery and postpartum information examined included gestational age at delivery, infant birth weight, route of delivery, the rate of postpartum hemorrhage and endometritis. Finally, fetal outcomes examined included the rate of small for gestational age (<10th percentile for respective gestation age according to the American Academy of Pediatrics [14]), low Apgars, NICU admission, respiratory distress syndrome (as documented in the pediatric note), perinatal HIV transmissions, and length of hospital stay.

For the purpose of this analysis preeclampsia was defined as at least two blood pressures of 140/90 six hours apart with proteinuria. Chorioamnionitis was defined as intrapartum fever ≥ 38°C alone or in conjunction with maternal leukocytosis, uterine tenderness, foul-smelling amniotic fluid, and maternal and/or fetal tachycardia. Anemia was noted if the hemoglobin was less than 11 mg/dL. Preterm labor was defined as contractions with cervical change at <37 weeks. Abruption was diagnosed via painful contractions and vaginal bleeding and confirmed on placental evaluation at the time of delivery. PPROM was diagnosed either by pooling on speculum exam or ferning on a dry slide with positive nitrazine. Postpartum hemorrhage was defined as >500 cc blood loss for vaginal delivery and >1000 cc for cesarean and finally endometritis was diagnosed when postpartum fever with fundal tenderness or malodorous vaginal discharge was noted.

Univariate analyses were conducted to evaluate differences between those pregnancies with and without a concomitant STI. Chi square and Fisher exact tests were used to analyze categorical variables. A* t*-test and Mann-Whitney *U* test were used to compare continuous variables. Odd ratios with 95% confidence intervals were calculated to examine associations between STIs and primary and secondary outcomes of interest. Multiple logistic regression analysis was performed to adjust for potential confounders. Statistical Package for the Social Sciences (SPSS V21) was used for all the analyses and statistical significance was defined as a *P* value < 0.05.

## 3. Results 

During the study period January 1, 2003, through May 31, 2013, HIV infected women delivered 414 pregnancies. Of these pregnancies, 266 (64%) were complicated by a concomitant STI diagnosed during their pregnancy. Of the women enrolled, STIs were diagnosed in the following: HPV 44.6% (*n* = 186), HSV 16.2% (*n* = 67), chlamydia 14.5% (*n* = 60), trichomoniasis 14.5% (*n* = 60), gonorrhea 4.1% (*n* = 17), and syphilis 2.4% (*n* = 10). Of the HIV infected women with concomitant STIs, most had one STI during their pregnancy (*n* = 165) 62%; however 22% (*n* = 59) of the women had two STIs, and 12% (*n* = 32) had three or more STIs. Fifty-six women (13.5%) contributed more than one pregnancy to the cohorts, and fourteen women (3.4%) contributed three or four pregnancies to the cohort. To screen for potential selection bias a sensitivity analysis in the subgroup with repeat pregnancies was conducted comparing the outcomes from all pregnancies to those of only the index pregnancy. The analysis revealed no difference in outcome between index and subsequent pregnancies.

Demographic and clinical characteristics of the study cohort are shown in Table 1. The majority of pregnancies occurred in multiparous black women who reported using antiretroviral medications.

Characteristics of the study population stratified by presence or absence of concomitant STIs are shown in Table 2. STI infected mothers compared to STI negative mothers were younger (*P* < 0.001), had a lower CD4 count at entry to prenatal care (*P* = 0.002), and were more likely to require antepartum admission. The two groups were comparable in body weight, race, gravidity, prior PTB, perinatal HIV transmissions, and years of HIV infection.

The effect of the acquisition of a concomitant STIs on perinatal and neonatal outcomes of interest are shown in Table 3. HIV infected women with concomitant STIs had a higher risk of spontaneous preterm birth (odds ratio (OR) 2.11, 95% confidence interval (CI), 1.12–3.97). In addition, these women also had a higher incidence of RDS being diagnosed in their infant (OR 3.72, 95% CI, 1.08–12.78). There was no difference in the risk of other perinatal and neonatal outcomes of interest examined. Stratified analysis based on the number of STIs revealed that women who had three or more concomitant STIs had an increased risk of spontaneous preterm birth (odd ratio 3.75, 95% confidence interval 1.45–9.66).

The results of multivariate analysis revealed that after adjusting for a history of PTB, maternal age, and low CD4 count (<200) at entry to prenatal care the increased risk for spontaneous preterm birth persisted (adjusted odds ratio 1.96, 95% confidence interval 1.01–3.78).

## 4. Discussion

The major finding of our study is that HIV infected women who acquire a concomitant STI during pregnancy had a 2-fold increased risk of spontaneous preterm delivery and likely therefore RDS. This increased risk persisted after adjusting for potential confounders including a history of preterm birth, maternal age, and low CD4+ count (<200) near the time of entry to prenatal care. Our results seem biologically plausible as even studies in pregnant HIV negative women report that acquisition of STIs during pregnancy is associated with increased risk for PTB [9, 10, 13]. Taken together the finding of an increased risk of preterm delivery in any woman with an STI suggests a potential contribution of STIs to adverse pregnancy outcomes. It is hypothesized that a potential mechanism for PTB in the setting of STIs may be mediated by inflammation with activation of cytokine cascade [7].

Our findings support the importance of routine STI screening in HIV infected women during pregnancy. Current practice guidelines for routine STI screening in pregnancy recommend screening at entry to prenatal care and, then in regards to chlamydia, women < 25 or at high risk for its acquisition should be screened again in the third trimester [7]. However, the results of our study suggests that there may be a role for routine second trimester STI screening in HIV infected women as well since the majority of STIs in our study were detected in the second trimester. In addition, to enhance STI screening it is important for health care providers to encourage condom use and safe sex practices in their HIV infected pregnant patients. Health care providers should educate patients on signs and symptoms of STIs during pregnancy. Women diagnosed with STIs need close follow-up and test of cures to ensure adequate treatment. Prevention and early treatment of STIs may decrease the risk of spontaneous preterm birth among HIV positive women.

Our study has several strengths. First, this study reports a well characterized cohort. HIV infected women were prospectively identified and data was collected on women who received prenatal care at the same hospital and by the same physicians. Second, this was a contemporary managed HIV infected population as over 95% of women in each cohort were maintained on combination antiretroviral therapy. Finally, multivariate analysis was performed to adjust for potential confounders that could influence the risk of spontaneous preterm birth.

Our study also has several limitations. A major limitation is that the diagnosis of HPV infection was presumptive and based on the presence of an abnormal pap test. Studies do however show a strong association (97–99%) between cervical dysplasia and the presence of HPV infection [15, 16]. Another limitation of our study is potential misclassification as women who had negative cytology and no STIs may have had HPV infection. However, this misclassification would be in the direction of the null hypothesis and should not have appreciably impacted our results. Finally, there may be unknown confounder that exists and were not controlled for in the multivariable analysis.

In conclusion, HIV infected women were found to have a 2-fold increased risk of spontaneous preterm birth when they were coinfected with an STI during pregnancy. Therefore enhanced STI screening with tests of cure, along with safe sex counseling, are vital steps in potentially mitigating adverse outcome in HIV infected pregnant women.

## Figures and Tables

**Table 1:** Key demographic and clinical characteristics of study population.

Characteristics	All pregnancies (*n* = 414)
Age (years) (*N* = 414)^*^	27.5 ± 6.2
Maternal wt. (lbs) (*n* = 292)^*^	162.3 ± 46.4
Race (*n* = 414)	
Black	355 (85.7%)
Hispanic	29 (7.0%)
White	20 (4.8%)
Asian	6 (1.4%)
Gravidity (*n* = 414)	
G1	78 (18.8%)
G2 or 3	189 (45.7%)
G4+	148 (35.7%)
Prior preterm birth (*n* = 414)	57 (13.8%)
Perinatally infected HIV positive mom	20 (5.0%)
Time since HIV infection diagnosis (*n* = 400)	
<1 year	65 (16.3%)
1–5 years	174 (43.5%)
>5–10	93 (23.3%)
>10 years	68 (17.0%)
CD4 count at entry (*n* = 359)^†^	352.0 (198.0, 564.0)
Using antiretroviral medications (*n* = 414)	401 (96.9%)
Duration of antiretroviral use (*n* = 414)	
Not using medications	13 (3.1%)
<1 month	18 (4.3%)
1–5 months	282 (68.1%)
6–12 months	74 (17.9%)
>12 months	27 (6.5%)

Data are number (%) unless otherwise specified.

^*^Mean ± SD, ^†^IQR-Interquartile range.

**Table 2:** Characteristics of HIV infected pregnancies with and without concomitant sexually transmitted infections.

Characteristics	STI+ (*n* = 266)	STI− (*n* = 148)	*P* value
Age (years)^*^	26.4 ± 6.1	29.5 ± 5.9	<0.001
Maternal weight (pounds)^*^	162.5 ± 48.2	159.9 ± 38.7	0.836^MW^
Race			
Black	229 (87.1%)	126 (85.7%)	0.893
Hispanic	18 (6.8%)	11 (7.5%)	
White	13 (4.9%)	7 (4.8%)	
Asian	3 (1.1%)	3 (2.0%)	
Gravidity			
G1	55 (20.3%)	23 (15.5%)	0.088
G2 or 3	127 (47.7%)	62 (41.9%)	
G4+	85 (32.0%)	63 (42.6%)	
Prior preterm birth	35 (13.2%)	22 (16.7%)	0.301
Perinatally infected HIV positive mom	14 (5.5%)	6 (4.2%)	0.566
Time since HIV Diagnosed			
<1 year	42 (15.8%)	22 (15.3%)	0.966
1–5 years	111 (41.7%)	61 (41.2%)	
>5–10	57 (21.4%)	34 (23.0%)	
>10 years	42 (15.8%)	25 (17.4%)	
CD4 Count at entry^*^	373.3 ± 272.2	456.8 ± 262.2	<0.001^MW^
Antepartum admission related to HIV infection	26 (11.9%)	5 (4.0%)	0.018
Using antiretroviral medications	256 (96.6%)	145 (98.6%)	0.341

Data are number (%) unless otherwise specified.

^*^Mean ± SD.

*P* values determined using *t*-test or MW-Mann-Whitney *U *test for continuous variables and Chi square or Fisher's exact for discrete variables.

**Table 3:** Perinatal and neonatal outcomes in HIV+ women with and without concomitant sexually transmitted infections.

Outcomes	STI+ *N* = 266	STI− *N* = 148	Total *N* = 414	OR 95% CI	*P* value
Preterm birth					
+	57 (21.4%)	21 (14.2%)	78 (18.8%)	1.65 (0.96–2.85)	0.073
−	209 (78.6%)	127 (85.8%)	336 (81.2%)		
Spontaneous preterm birth					
+	48 (18.0%)	14 (9.5%)	62 (15.0%)	2.11 (1.12–3.97)	0.021
−	218 (82.0%)	134 (90.5%)	352 (85.0%)		
Indicated preterm birth					
+	9 (50.0%)	7 (50.0%)	16 (50.0%)	1.00 (0.25–4.04)	1.00
−	9 (50.0%)	7 (50.0%)	16 (50.0%)		
Postpartum hemorrhage					
+	20 (7.9%)	8 (5.5%)	28 (7.0%)	1.47 (0.63–3.44)	0.369
−	234 (92.1%)	138 (94.5%)	372 (93.0%)		
Chorioamnionitis					
+	10 (3.9%)	2 (1.4%)	12 (3.0%)	2.91 (0.63–13.45)	0.172
−	246 (96.1%)	143 (98.6%)	389 (97.0%)		
Endometritis					
+	12 (4.5%)	3 (2.0%)	15 (3.6%)	2.28 (0.63–8.22)	0.207
−	254 (95.5%)	145 (98%)	399 (96.4%)		
Preeclampsia					
+	13 (5.0%)	10 (6.8%)	23 (5.7%)	0.73 (0.31–1.71)	0.467
−	246 (95.0%)	138 (93.2%)	384 (94.3%)		
Diabetes					
+	10 (3.8%)	6 (4.1%)	16 (3.9%)	0.94 (0.33–2.64)	0.905
−	252 (96.2%)	142 (95.9%)	394 (96.1%)		
IUGR					
+	6 (2.3%)	1 (0.7%)	7 (1.7%)	3.41 (0.41–28.62)	0.258
−	255 (97.7%)	145 (99.3%)	400 (98.3%)		
Small for gestational age					
+	23 (8.7%)	12 (8.1%)	35 (8.5%)	1.08 (0.52–2.24)	0.833
−	241 (91.3%)	136 (91.9%)	377 (91.5%)		
1 min Apgar <7					
+	33 (12.7%)	19 (12.9%)	52 (12.8%)	0.98 (0.53–1.79)	0.946
−	227 (87.3%)	128 (87.1%)	355 (87.2%)		
5 min Apgar <7					
+	33 (12.7%)	19 (12.9%)	52 (12.8%)	0.98 (0.53–1.79)	0.946
−	227 (87.3%)	128 (87.1%)	355 (87.2%)		
NICU admission					
+	52 (20.6%)	21 (14.6%)	73 (18.4%)	1.52 (0.87–2.65)	0.137
−	200 (79.4%)	123 (85.4%)	323 (81.7%)		
RDS					
+	19 (7.1%)	3 (0.7%)	22 (5.3%)	3.72 (1.08–12.78)	0.037
−	247 (92.9%)	145 (99.3%)	392 (94.7%)		
Infant HIV					
+	3 (1.1%)	3 (2.0%)	6 (1.4%)	0.55 (0.11–2.77)	0.469
−	263 (98.9%)	145 (98.0%)	408 (98.6%)		

IUGR-intrauterine growth restriction; NICU-neonatal intensive care unit; RDS-respiratory distress syndrome (as documented in pediatric note)

OR odds ratio, CI confidence interval.
